# Sex-specific differences in myocardial glucose metabolic rate in non-diabetic, pre-diabetic and type 2 diabetic subjects

**DOI:** 10.1186/s12933-024-02246-7

**Published:** 2024-04-26

**Authors:** Elena Succurro, Patrizia Vizza, Francesco Cicone, Velia Cassano, Mattia Massimino, Federica Giofrè, Teresa Vanessa Fiorentino, Maria Perticone, Angela Sciacqua, Pietro Hiram Guzzi, Pierangelo Veltri, Francesco Andreozzi, Giuseppe Lucio Cascini, Giorgio Sesti

**Affiliations:** 1 Department of Medical and Surgical Sciences, University Magna Graecia of Catanzaro, Viale Europa, 88100 Catanzaro, Italy; 2 Research Center for the Prevention and Treatment of Metabolic Diseases (CR METDIS), University Magna Graecia of Catanzaro, Catanzaro, Italy; 3 Department of Experimental and Clinical Medicine, Magna Graecia University of Catanzaro, Catanzaro, Italy; 4 Department of Computer Engineering, Electronics and Systems, University of Calabria, ModelingRende, Italy; 5 Department of Clinical and Molecular Medicine, University of Rome-Sapienza, 00189 Rome, Italy

**Keywords:** Sex-differences, Myocardial glucose metabolism, Cardiovascular disease, Type 2 diabetes, Prediabetes, Cardiac ^18^F-FDG PET

## Abstract

**Background:**

Evidence has shown that women with type 2 diabetes (T2DM) have a higher excess risk for cardiovascular disease (CVD) than men with T2DM. Subjects with either T2DM or prediabetes exhibit myocardial insulin resistance, but it is still unsettled whether sex-related differences in myocardial insulin resistance occur in diabetic and prediabetic subjects.

**Methods:**

We aimed to evaluate sex-related differences in myocardial glucose metabolic rate (MRGlu), assessed using dynamic PET with ^18^F-FDG combined with euglycemic-hyperinsulinemic clamp, in subjects with normal glucose tolerance (NGT; n = 20), prediabetes (n = 11), and T2DM (n = 26).

**Results:**

Women with prediabetes or T2DM exhibited greater relative differences in myocardial MRGlu than men with prediabetes or T2DM when compared with their NGT counterparts. As compared with women with NGT, those with prediabetes exhibited an age-adjusted 35% lower myocardial MRGlu value (P = 0.04) and women with T2DM a 74% lower value (P = 0.006), respectively. Conversely, as compared with men with NGT, men with T2DM exhibited a 40% lower myocardial MRGlu value (P = 0.004), while no significant difference was observed between men with NGT and prediabetes. The statistical test for interaction between sex and glucose tolerance on myocardial MRGlu (P < 0.0001) was significant suggesting a sex-specific association.

**Conclusions:**

Our data suggest that deterioration of glucose homeostasis in women is associated with a greater impairment in myocardial glucose metabolism as compared with men. The sex-specific myocardial insulin resistance could be an important factor responsible for the greater effect of T2DM on the excess risk of cardiovascular disease in women than in men.

## Introduction

Cardiovascular disease (CVD) represents the leading causes of death worldwide both in men and women [1]. In 2021, the International Diabetes Federation (IDF) Atlas estimated that 2.30 million cardiovascular deaths and 5.4 million deaths overall were attributable to elevated fasting plasma glucose [2, 3]. Indeed, data from large longitudinal studies have shown that the risk for major cardiovascular events is 2–3 times higher in individual with type 2 diabetes (T2DM) as compared to people without diabetes [2, 4–10].

Although men have a higher absolute risk of CVD, several studies have shown that women with T2DM have an excess risk of CV events, including coronary heart disease and stroke as compared with men [5–10]. The factors contributing to sex-related differences in relative risk of CV events is still a subject of debate. It has been suggested that deterioration in glucose homeostasis from normal glucose tolerance to prediabetes to overt T2DM is associated with worsening in metabolic risk profile and target organ damage in women than men [5, 11–15]. CV risk factors progress during menopausal transition, determining greater accumulation of fat in visceral and ectopic tissues and aggravating insulin resistance, inflammation and dyslipidemia in women with T2DM [16]. Moreover, it has been hypothesized that hyperglycemia has a stronger effect on CV risk factors in women than men [5, 11–16].

It has been reported that subjects with T2DM and individuals at increased risk of T2DM, including those with prediabetes and metabolic syndrome, exhibit myocardial insulin resistance, considered an independent predictor of CV events [17–24]. Impaired insulin-stimulated myocardial glucose metabolism has been associated with reduced myocardial mechano-energetic efficiency, and increased cardiac workload [24, 25], both alterations linked to the development of heart failure and CV events [26, 27]. Furthermore, a reduced myocardial glucose uptake has been associated with carotid, aortic and coronary atherosclerosis [23, 24, 28]. Additionally, there is consistent evidence that myocardial blood flow reserve assessed by Positron Emission Tomography (PET) is impaired in patients with T2DM [29]. A reduction of coronary flow reserve has been directly associated with severity of stenosis in patients with coronary heart disease [29].

However, it is still unsettled whether sex-related differences in myocardial insulin resistance occur in diabetic and prediabetic subjects.

In light of the significant pathophysiological role of myocardial insulin resistance, the primary aim of this study was to determine whether sex-related differences exist in insulin-stimulated myocardial glucose metabolic rate (MRGlu) using dynamic myocardial PET with ^18^F-Fluorodeoxyglucose (^18^F-FDG) combined with euglycemic-hyperinsulinemic clamp, in individuals with normal glucose tolerance, prediabetes and type 2 diabetes without history of heart disease.

## Methods

### Study participants

The study cohort comprised 57 subjects participating in the CATAnzaro MEtabolic RIsk factors (CATAMERI) study, an observational study recruiting adult individuals bearing one or more cardio-metabolic risk factors, and consecutively recruited at a referral university hospital of the University “Magna Graecia” of Catanzaro [18, 22]. The participants to the present study were randomly selected from the CATAMERI cohort to perform a state-of-art characterization of insulin-stimulated myocardial glucose metabolism using cardiac ^18^F-FDG-PET scan combined with euglycemic hyperinsulinemic clamp technique [18, 22]. Eligible subjects were recruited according to the following inclusion criteria: age between 30 and 70 years, and positivity for one or more cardio-metabolic risk factors including family history of diabetes, impaired fasting glucose, hypertension, dyslipidemia, and overweight/obesity. Exclusion criteria were type 1 diabetes, end-stage renal disease, previous CVD on the basis of medical history, resting electrocardiogram and stress test or myocardial scintigraphy for individuals with T2DM, history of atrial fibrillation or other arrhythmias, right and left bundle branch block, dyssynchrony in ventricular contraction, valvular heart disease, liver cirrhosis, history of malignant or autoimmune diseases, acute or chronic infections, history of alcohol or drug abuse and treatment with drugs known to influence glucose tolerance such as steroids and estro-progestins and medicaments affecting heart function including beta blockers and antiarrhythmic drugs. All subjects underwent a comprehensive screening for cardiovascular complications, including electrocardiogram, echocardiogram, as previously described [12, 18]. All subjects underwent anthropometrical evaluation including measurements of body mass index (BMI), waist circumference and body composition by bioelectrical impedance, and assessment of whole-body and cardiac insulin sensitivity. Readings of clinic blood pressure (BP) were measured at 3-min intervals using a standard sphygmomanometer, and BP values were the average of 3 measurements after a 10-min period of rest in the supine position. After an overnight fasting, biochemical determinations and a 75 g OGTT were performed in individuals with FPG < 126 mg/dl, HbA1c < 6.5% and no history of T2DM. According to the ADA criteria [30], individuals were classified as having normal glucose tolerance (NGT) when fasting plasma glucose was < 100 mg/dl (5.5 mmol/l), 2-h postload glucose < 140 mg/dl (< 7.77 mmol/l) and HbA1c < 5.7%, prediabetes when fasting plasma glucose was 100–125 mg/dl (5.5–6.9 mmol/l), 2-h postload glucose 140–199 mg/dl (7.77–11.0 mmol/l) or HbA1c 5.7–6.4%, T2DM when fasting plasma glucose was ≥ 126 mg/dl (> 7 mmol/l), 2-h post-load glucose was ≥ 200 mg/dl (> 11.1 mmol/l), HbA1c ≥ 6.5% or in treatment with antidiabetic drugs.

On the second day, after 12-h fasting, all subjects underwent ^18^F-FDG PET scan combined with euglycemic hyperinsulinemic clamp. Subjects with T2DM took their last dose of metformin during the previous dinner, 12 h before the study.

The study was approved by the Ethics Committee (Comitato Etico Azienda Ospedaliera “Mater Domini”), and informed consent was obtained from each subject in accordance with principles of the Declaration of Helsinki.

### ^***18***^***F-FDG PET scan combined with euglycemic hyperinsulinemic clamp***

Myocardial glucose metabolic rate (MRGlu), expressed as μmol/min/100 g, was measured by ^18^F-FDG-PET acquired during an euglycemic hyperinsulinemic clamp as previously described [24]. Subjects received a priming dose of insulin (100 UI/mL) (Humulin R; Eli Lilly) during the initial 10 min to raise the serum insulin concentration acutely (80 mU/m^2^ body surface area × min), and then it was maintained by continuous insulin infusion fixed at 40 mU/m2 body surface area × min [31]. The blood glucose level was maintained constant at 90 mg/dl for the next 120 min by infusing 20% glucose at varying rates according to blood glucose measurements performed at 5-min intervals (mean coefficient of variation of blood glucose was < 4%). For individuals with T2DM the blood glucose level was maintained constant at 100 mg/dl for the next 120 min by infusing 20% glucose at varying rates according to blood glucose measurements performed at 5-min intervals (mean coefficient of variation of blood glucose was < 4%). Glucose metabolized by the whole body (M) was calculated as the mean rate of glucose infusion measured during the last 60 min of the clamp examination (steady state) and was expressed as milligrams per minute per kilogram fat-free mass (M_FFM_).

The ^18^F-FDG-PET imaging procedure was performed on a hybrid PET/CT scanner (GE Discovery ST8-2D PET scanner), starting 60 min after the insulin infusion. A 60-min dynamic acquisition was started simultaneously with the intravenous injection of 370 MBq^18^F-FDG, according to the following time frame sampling: 8 × 15 s, 2 × 30 s, 2 × 120 s, 1 × 180 s, 6 × 300 s, 2 × 600 s [32]. PET images were reconstructed in a 128 × 128 matrix using a OSEM algorithm, and corrected for decay and attenuation based on co-registered CT. Hounsfild units (HU) are extracted from CT images, then they are used to correct the corresponding voxel of PET images. This process is named attenuation correction. The insulin-glucose infusion continued during the entire PET acquisition. The estimation of myocardial MRGlu was performed by Patlak compartmental modelling [33], using the graphical tool specific for cardiac images analysis (PCARD) implemented in PMOD Software platform (Version 3.806) [32]. In PCARD, the full dynamic study is used for MRGlu calculation, and the arterial input function is extracted from a volume of interest (VOI) semi-automatically placed in the left ventricular cavity [33].

### Laboratory determinations

Plasma glucose, total and HDL cholesterol, and triglycerides were assayed using enzymatic methods (Roche Diagnostics, Mannheim, Germany). HbA1c was measured with high performance liquid chromatography using an NGSP-certified automated analyzer (Adams HA-8160 HbA1c analyzer, Menarini, Italy).

### Statistical analyses

Variables with skewed distribution including triglycerides were natural log transformed for statistical analyses. Continuous variables are expressed as means ± SD. Categorical variables were compared by χ^2^ test. Comparisons between women and men were performed using unpaired Student’s t test. A general linear model with post hoc Bonferroni correction for multiple comparisons was used to compare differences of continuous variables between groups. Comparisons between NGT, prediabetes and T2DM groups were performed separately in men and women using a general linear model with post hoc Fisher's least significant difference correction for pairwise comparisons. Statistical interactions for sex by glucose tolerance categories in their association with both myocardial glucose metabolic rate and insulin-stimulated glucose disposal were computed using general linear model to determine whether changes in means among subjects with different glucose tolerance status differed by sex.

Previous studies have shown a 40% decrease of myocardial glucose metabolism rate in subjects with IGT compared to those with normal glucose tolerance [34]. Considering mean value of myocardial glucose metabolic rate of 28.3 ± 5 μmol/min/100 g for subjects with normal glucose tolerance and a mean value of 18.6 μmol/min/100 g in subjects with IGT, a two-sample t test with a two-sided significance level of 0.05 and power of 0.80 results in a sample size of 4 per group as computed using an online power calculator (https://clincalc.com/stats/samplesize.aspx).

For all analyses a P value < 0.05 was considered to be statistically significant. All analyses were performed using SPSS software Version 29 for Mac.

## Results

### Differences in anthropometric and cardiovascular features between subjects with NGT, prediabetes and T2DM according to sex

Study population comprised 57 subjects, of whom 27 (47.4%) were female, and 30 (52.6%) were male. Of the 27 recruited women, 12 (44.4%) had NGT, 7 (26%) had prediabetes, 8 (29.6%) had T2DM. Of the 30 recruited men, 8 (26.6%) had NGT, 4 (13.3%) had prediabetes, 18 (60.1%) had T2DM. Anthropometric and cardiovascular features of individuals with NGT, prediabetes and T2DM according to sex are shown in Table 1. All the subjects with type 2 diabetes were treated with metformin with a mean dosage of 2306 mg and 2347 mg, for women and men, respectively.

**Table 1:** Differences in clinical characteristics of women and men with NGT, prediabetes, and T2DM

	NGT			Prediabetes			T2DM		
	Women (n = 12)	Men (n = 8)	P values	Women (n = 7)	Men (n = 4)	P value	Women (n = 8)	Men (n = 18)	P value
Age (yrs)	42 ± 8	46 ± 11	0.3	49 ± 13	55 ± 5	0.3	57 ± 2	54 ± 8	0.4
BMI (kg/m^2^)	28.5 ± 5.9	29.5 ± 3.8	0.7	30.5 ± 7	25 ± 0.6	0.1	31.2 ± 5.4	31.3 ± 3.8	0.9
Waist circumference (cm)	96.5 ± 13	104 ± 9	0.1	99.5 ± 17	95 ± 6	0.6	104.7 ± 11	108 ± 8	0.3
Systolic blood pressure (mmHg)	112 ± 16	121 ± 19	0.2	129 ± 10	127 ± 18	0.8	125 ± 11	127 ± 13	0.7
Diastolic blood pressure (mmHg)	71 ± 10	76 ± 12	0.4	76 ± 9	74 ± 11	0.7	73 ± 10	81 ± 10	0.09
Heart rate (beats min^−1^)	66 ± 4	69 ± 10	0.2	72 ± 11	68 ± 14	0.5	75 ± 9	73 ± 6	0.4
Total cholesterol (mg/dl)	194 ± 47	193 ± 29	0.9	200 ± 38	206 ± 27	0.7	161 ± 22	188 ± 40	0.09
HDL cholesterol(mg/dl)	57 ± 11	45 ± 12	0.04	51 ± 6	41 ± 8	0.04	48 ± 7	41 ± 8	0.05
LDL cholesterol (mg/dl)	125 ± 39	125 ± 36	0.9	136 ± 41	141 ± 22	0.8	108 ± 24	120 ± 30	0.3
Triglycerides (mg/dl)	117 ± 83	133 ± 75	0.6	118 ± 34	130 ± 53	0.6	156 ± 59	142 ± 77	0.9
Fasting Plasma Glucose (mg/dl)	88 ± 6	85 ± 6	0.3	94 ± 19	99 ± 8	0.6	150 ± 37	140 ± 40	0.5
2-h post load plasma glucose (mg/dl)	111 ± 13	121 ± 14	0.1	149 ± 22	142 ± 1	0.5	–	–	–
Fasting Plasma Insulin (mU/mL)	12.5 ± 5.8	11.5 ± 4.2	0.3	14.2 ± 13	8.4 ± 3.5	0.1	11.1 ± 8	13.6 ± 7.9	0.2
HbA1c (%)	5.5 ± 0.4	5.4 ± 0.6	0.5	5.7 ± 0.3	5.9 ± 0.2	0.5	7.8 ± 0.9	7.4 ± 1.1	0.3
Fat mass (Kg)	24.2 ± 10	23.2 ± 5.2	0.4	26.8 ± 11	18.9 ± 1.8	0.09	25.3 ± 12	26.3 ± 5.7	0.4
Fat free mass (Kg)	45.7 ± 4.3	44.4 ± 6.1	0.1	47.1 ± 5.4	58.4 ± 2.1	0.01	46.8 ± 5.0	46.7 ± 8.0	0.9
Fasting plasma glucose concentration at the beginning of the clamp (mg/dl)	80.4 ± 6.2	81.4 ± 15	0.3	78.1 ± 13	83.2 ± 20	0.4	128 ± 25	121.5 ± 24	0.2
Antihypertensive therapy (%)	8.3	37.5	0.21	14.3	25	0.1	62.5	66.7	0.79
Lipid-lowering therapy (%)	8.3	12.5	0.7	14.3	25	0.06	75	44.4	0.45
Glucose-lowering therapy	–	–	–	–	–	–	100	100	0.9
Meftormin (%)	–	–	–	–	–		100	100	0.9
Diabetes duration (years)	–	–	–	–	–	–	5.2 ± 4.0	3.9 ± 3.0	0.1
Whole body insulin-stimulated glucose disposal (mg/min × Kg FFM)	9.58 ± 8.3	6.9 ± 6.1	0.4	3.74 ± 1.6	5.6 ± 5.5	0.3	2.54 ± 2.7	3.02 ± 1.7	0.5
Myocardial MrGlu (μmol/min/100 g)	25.7 ± 6.9	28.3 ± 5.4	0.4	16.7 ± 7.2	18.6 ± 6.4	0.6	6.6 ± 7.01	16.9 ± 10.1	0.02

Data are means ± SD, unless otherwise indicated. Categorical variables were compared by χ2 test. Comparisons between women and men were performed using unpaired Student’s t test. ^§^P values refer to results after analyses with adjustment for age. Triglycerides levels were log transformed for statistical analysis, but values in the table represent a back transformation to the original scale

No sex-related differences in age, anthropometric, and cardiometabolic features were observed across the three glucose tolerance categories, except for levels of HDL cholesterol which were significantly lower in men as compared with women in all three glucose tolerance categories (Table 1). In addition, as compared with women with prediabetes, men showed a higher FFM (Table 1).

### Differences in myocardial glucose metabolic rate and insulin sensitivity between subjects with NGT, prediabetes and T2DM according to sex

Women with NGT exhibited a numerically higher, although not statistically significant, mean value of insulin-stimulated glucose disposal as compared with men with NGT. No sex-related differences in whole-body insulin-stimulated glucose disposal were observed between men and women in prediabetes and diabetic groups (Table 1). No sex-related differences in myocardial glucose metabolic rate were observed in NGT and prediabetes groups (Table 1). Conversely, among T2DM patients, women exhibited a significant lower myocardial glucose metabolic rate value than men (Table 1).

### Age-adjusted differences in cardiovascular risk factors between women with NGT, prediabetes and T2DM

As compared with women with NGT, those with T2DM were older, and showed higher age-adjusted fasting plasma glucose, HbA1c and fasting glucose at the beginning of the euglycemic hyperinsulinemic clamp (Table 2). Furthermore, women with prediabetes exhibited higher age-adjusted 2-h post load plasma glucose levels. Moreover, women with T2DM were significantly more likely to be treated with lipid-lowering therapy and metformin as compared with those with NGT (Table 2).

**Table 2:** Differences in clinical characteristics of women and men with NGT, prediabetes, and T2DM

	Women			Prediabetes vs. NGT	T2DM vs. NGT	Men			Prediabetes vs. NGT	T2DM vs. NGT
	NGT (n = 12)	Prediabetes (n = 7)	T2DM (n = 8)	P value	P value	NGT (n = 8)	Prediabetes (n = 4)	T2DM (n = 18)	P value	P value
Age (yrs)	42 ± 8	49 ± 13	57 ± 2	0.5	0.01	46 ± 11	55 ± 5	54 ± 8	0.3	0.1
BMI (kg/m^2^)	28.5 ± 5.9	30.5 ± 7	31.2 ± 5.4	0.8^§^	0.3^§^	29.5 ± 3.8	25 ± 0.6	31.3 ± 3.8	0.1^§^	0.02^§^
Waist circumference (cm)	96.5 ± 13	99.5 ± 17	104.7 ± 11	0.9^§^	0.2^§^	104 ± 9	95 ± 6	108 ± 8	0.1^§^	0.06^§^
Systolic blood pressure (mmHg)	112 ± 16	129 ± 10	125 ± 11	0.09^§^	0.8^§^	121 ± 19	127 ± 18	127 ± 13	0.1^§^	0.01^§^
Diastolic blood pressure (mmHg)	71 ± 10	76 ± 9	73 ± 10	0.9^§^	0.5^§^	76 ± 12	74 ± 11	81 ± 10	0.4^§^	0.2^§^
Heart rate (beats min^−1^)	66 ± 4	72 ± 11	75 ± 9	0.056^§^	0.09^§^	69 ± 10	68 ± 14	73 ± 6	0.9^§^	0.03^§^
Total cholesterol (mg/dl)	194 ± 47	200 ± 38	161 ± 22	0.5^§^	0.07^§^	193 ± 29	206 ± 27	188 ± 40	0.5^§^	0.7^§^
HDL (mg/dl)	57 ± 11	51 ± 6	48 ± 7	0.4^§^	0.3^§^	45 ± 12	41 ± 8	41 ± 8	0.6^§^	0.2^§^
LDL cholesterol (mg/dl)	125 ± 39	136 ± 41	108 ± 24	0.8^§^	0.1^§^	125 ± 36	141 ± 22	120 ± 30	0.3^§^	0.9^§^
Triglycerides (mg/dl)	117 ± 83	118 ± 34	156 ± 59	0.5^§^	0.8^§^	133 ± 75	130 ± 53	142 ± 77	0.7^§^	0.5^§^
Fasting plasma Glucose (mg/dL)	88 ± 6	94 ± 19	150 ± 37	0.9^§^	< 0.0001^§^	85 ± 6	99 ± 8	140 ± 40	0.01^§^	0.002^§^
2-h post load plasma glucose (mg/dl)	111 ± 13	149 ± 22	–	< 0.0001^§^	–	121 ± 14	142 ± 1	–	0.053	–
Fasting plasma insulin (mU/mL)	12.5 ± 5.8	14.2 ± 13	11.1 ± 8	0.2^§^	0.1^§^	11.5 ± 4.2	8.4 ± 3.5	13.6 ± 7.9	0.4^§^	0.2^§^
HbA1c (%)	5.5 ± 0.4	5.7 ± 0.3	7.8 ± 0.9	0.9^§^	< 0.0001^§^	5.4 ± 0.6	5.9 ± 0.2	7.4 ± 1.1	0.3^§^	< 0.0001^§^
Fat mass (Kg)	24.2 ± 10	26.8 ± 11	25.3 ± 12	0.9^§^	0.8^§^	23.2 ± 5.2	18.9 ± 1.8	26.3 ± 5.7	0.03^§^	0.8^§^
Fat free mass (Kg)	45.7 ± 4.3	47.1 ± 5.4	46.8 ± 5.0	0.6^§^	0.3^§^	44.4 ± 6.1	58.4 ± 2.1	46.7 ± 8.0	0.4^§^	0.1^§^
Fasting plasma glucose concentration at the beginning of the clamp (mg/dl)	80.4 ± 6.2	81.4 ± 15	128 ± 25	0.8^§^	0.001^§^	78.1 ± 13	83.2 ± 20	121.5 ± 24	0.8^§^	0.001^§^
Antihypertensive therapy (%)	8.3	14.3	62.5	0.7^§^	0.4^§^	37.5	25	66.7	0.5^§^	0.4^§^
Lipid-lowering therapy (%)	8.3	14.3	75	0.7^§^	0.005^§^	12.5	25	44.4	0.5^§^	0.2^§^
Glucose-lowering therapy	–	–	100	–	< 0.0001^§^	–	–	100	–	< 0.0001^§^
Meftormin (%)	–	–	100	–	< 0.0001^§^	–	–	100	–	< 0.0001^§^
Diabetes duration (years)	–	–	5.2 ± 4.0	–	< 0.0001^§^	–	–	3.9 ± 3.0	–	< 0.0001^§^
Whole body insulin-stimulated glucose disposal (mg/min × Kg FFM)	9.58 ± 8.3	3.74 ± 1.6	2.54 ± 2.7	0.2^§^	0.02^§^	6.9 ± 6.1	5.6 ± 5.5	3.02 ± 1.7	0.6^§^	0.02^§^
Myocardial MrGlu (μmol/min/100 g)	25.7 ± 6.9	16.7 ± 7.2	6.6 ± 7.01	0.04^§^	0.006^§^	28.3 ± 5.4	18.6 ± 6.4	16.9 ± 10.1	0.02^§^	0.004^§^

Data are means ± SD, unless otherwise indicated. Categorical variables were compared by χ^2^ test. Comparisons between the three groups of glucose tolerance were performed using a general linear model with post hoc Fisher’s least significant difference correction for pairwise comparisons and post hoc Bonferroni correction for multiple comparisons. ^§^P values refer to results after analyses with adjustment for age. Triglycerides levels were log transformed for statistical analysis, but values in the table represent a back transformation to the original scale

### Age-adjusted differences in cardiovascular risk factors between men with NGT, prediabetes and T2DM

As compared with men with NGT, both those with prediabetes and T2DM were older, and showed higher age-adjusted fasting plasma glucose values (Table 2). Furthermore, as compared with men with NGT, those with prediabetes showed a significantly lower age-adjusted fat mass, whereas differences in BMI, waist circumference, and fat free mass were numerically but not statistically significant. Conversely, as compared with men with NGT, only those with T2DM showed higher age-adjusted BMI, systolic blood pressure, heart rate, HbA1c and fasting glucose at the beginning of the euglycemic hyperinsulinemic clamp (Table 2). No significant age-adjusted differences were observed in other cardiovascular risk factors among men with different glucose tolerance status.

### Age-adjusted differences in myocardial glucose metabolic rate and insulin sensitivity between women with NGT, prediabetes and T2DM

Age-adjusted myocardial glucose metabolic rate was significantly lower in women with prediabetes (P = 0.04), and T2DM (*P* = 0.006) as compared with those with NGT (Table 2). These differences remained also significant after further adjustment for Fasting Plasma Glucose and HbA1c (P = 0.004 for women with prediabetes, P = 0.01 for those with T2DM, respectively) and for women with T2DM also after further adjustment for antidiabetic and antihypertensive therapy (P = 0.009). Conversely, as compared with women with NGT, only women with T2DM exhibited a significant (*P* = 0.02) age-adjusted lower whole-body insulin-stimulated glucose disposal value, whereas those with prediabetes exhibited a numerically lower mean value, although not statistically significant (Table 2).

### Age-adjusted differences in myocardial glucose metabolic rate and insulin sensitivity between men with NGT, prediabetes and T2DM

As compared with men with NGT, age-adjusted myocardial glucose metabolic rate was significantly lower in men with prediabetes (P = 0.02), and T2DM (*P* = 0.004) as compared with those with NGT (Table 1). These differences were lost after further adjustment for glycemic parameters and also after further adjustment for antidiabetic and antihypertensive therapy. Conversely, as compared with men with NGT, age-adjusted whole-body insulin-stimulated glucose disposal was significantly lower in men with T2DM, whereas differences with individuals with prediabetes did not reach the statistically significant threshold likely due to small sample size (Table 2).

**Fig. 1:**
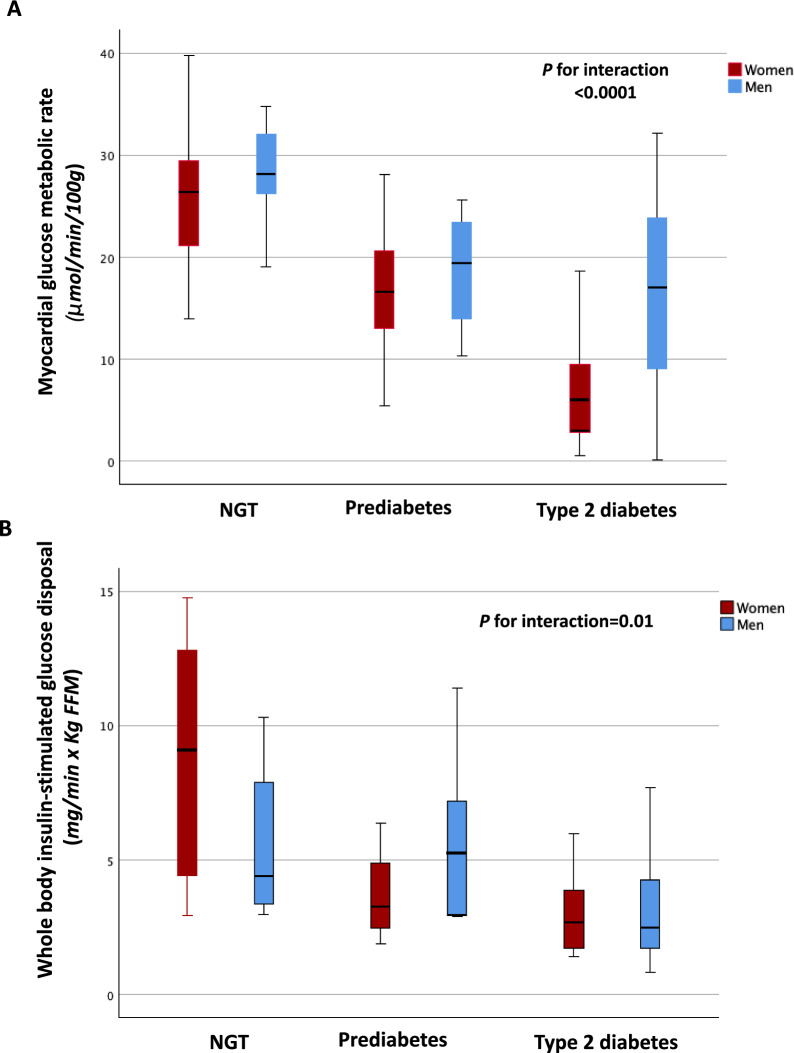
The estimated marginal means of cardiovascular variables according to sex and glucose tolerance status. **A** Myocardial glucose metabolic rate; **B** whole-body insulin-stimulated glucose disposal

The estimated marginal means of myocardial glucose metabolic rate and whole-body insulin-stimulated glucose according to sex and glucose tolerance status are reported in Fig. 1. Prediabetic and T2DM women exhibited greater relative differences in myocardial glucose metabolism and whole-body insulin-stimulated glucose disposal, than prediabetic and diabetic men when compared with their NGT counterparts. Formal tests for glucose tolerance status × sex interaction were statistically significant for myocardial glucose metabolic rate (P < 0.0001), and whole-body insulin-stimulated glucose disposal (P = 0.01) (Fig. 1).

## Discussion

To the best of our knowledge, the present study was the first to examine sex-related differences in myocardial glucose metabolism in individuals having different degrees of glucose tolerance. We found that deterioration in glucose homeostasis from normal glucose tolerance to prediabetes to T2DM was associated with a greater impairment in myocardial glucose metabolic rate, assessed using dynamic PET with ^18^F-FDG combined with euglycemic-hyperinsulinemic clamp, in women than men without coronary heart disease. Notably, as compared with women with NGT, those with prediabetes exhibited a 35% lower (P = 0.04) insulin-stimulated myocardial glucose metabolism and women with T2DM a 74% lower value (P = 0.006), respectively. Conversely, as compared with men with NGT, only men with T2DM exhibited a 40% lower myocardial glucose uptake (P = 0.004), while no significant difference was observed between men with NGT and prediabetes. In women, but not in men, these differences in myocardial glucose metabolism remained also significant after further adjustment for glycemic control.

Additionally, we found that prediabetic and T2DM women exhibited greater relative differences in whole-body insulin-stimulated glucose disposal, than prediabetic and diabetic men when compared with their NGT counterparts. These findings confirm and extend the notion that deterioration of glucose homeostasis in women is associated with a worsening in insulin sensitivity as compared with men [11, 14, 35]. In keeping with the present data, a previous study reported a greater impairment in insulin sensitivity assessed by euglycemic hyperinsulinemic clamp in women with prediabetes than men when compared with their NGT counterparts [14]. Accordingly, other studies have examined sex-related differences in insulin sensitivity using indices derived from oral glucose tolerance test (OGTT) in individuals having different degree of glucose tolerance showing that sex advantage in glucose metabolism seen in women with NGT vanished in T2DM [11, 36].

Growing evidence has shown that women with T2DM have a considerably higher diabetes-related relative risk for the incident of major CV events and mortality, including a 44% higher relative risk for coronary heart disease event, as compared with men [5–10]. We found a significant reduction in myocardial glucose metabolic rate in women with T2DM than male counterparts. An impairment in insulin-stimulated myocardial glucose metabolism in women with T2DM may indicate an early stage of myocardial energy disarrangement and reduced cardiac mechano-energetic performance [22, 23]. Indeed, lower myocardial glucose metabolic rate has been associated with a worse CV risk profile [23], and carotid and coronary atherosclerosis [23, 24, 28]. Furthermore, we have previously shown that as compared with men with T2DM, women with T2DM exhibited a higher reduction in cardiac mechano-energetic efficiency, and an increase in left ventricular mass, both being predictors of CV events [12, 26, 27, 37, 38]. Overall, the higher degree of myocardial insulin resistance observed in women with the altered glucose homeostasis as compared with men may be an early metabolic trigger leading to subsequent maladaptive changes involving cardiac geometry, myocardial mechanical energy efficiency, and coronary atherosclerosis, thus eventually ensuing in excess CV risk in women compared to men when diagnostic threshold of T2DM is reached. Clearly, further studies are warranted to confirm the role of myocardial insulin resistance in the sex-related differences in the development of CVD in T2DM.

The present findings are in agreement with those of previous studies showing the mechanisms of SGLT2 inhibitors on myocardial glucose metabolism assessed by PET with ^18^F-FDG combined with euglycemic-hyperinsulinemic clamp [39–41]. A previous study showed that 4-week treatment with dapagliflozin reduced epicardial adipose tissue glucose uptake in patients with T2DM and stable coronary artery disease improving myocardial flow reserve [39]. In line with this report, we previously demonstrated that treatment for 24 weeks with empagliflozin in patients with T2DM without coronary heart disease was associated with an improvement of myocardial insulin resistance, myocardial mechano-energetic efficiency, and cardiac function [40]. Similarly, another randomized study showed that 6-week treatment with dapagliflozin in patients with T2DM without heart failure reduced myocardial oxygen consumption [41].

The current results should be interpreted within the context of its strengths and limitations. A main strength is the use of gold standard methods to assess myocardial glucose metabolism by cardiac ^18^F-FDG PET scan combined with euglycemic hyperinsulinemic clamp technique, which allows the valuation of insulin-stimulated myocardial glucose uptake under uniform experimental conditions of euglycemia and physiological hyperinsulinemia [17, 42]. Moreover, glucose tolerance was accurately assessed using FPG, 2 h post-load glucose levels during an OGTT, and HbA1c according to ADA criteria thus excluding any potential misclassification of participants [30]. Additionally, all tests including ^18^F-FGD PET scan combined with euglycemic hyperinsulinemic clamp were collected by skilled examiners after a standardized training, who were blinded to the clinical data of the study participants.

Nonetheless, the present study also has some limitations. The results are only based on White individuals aging between 30 and 70 years thus limiting the generalizability of the present data to other ethnicities or to older individuals. Furthermore, the overall sample size was small and might produce misleading results, mostly in the group of subjects with prediabetes. . Third, all subjects with T2DM were treated with metformin monotherapy and were not subjects with newly diagnosed T2DM. Additionally, the cross-sectional design of the study precludes causal inferences, and, therefore, no conclusions regarding cause-effect relationships can be made.

## Conclusions

In conclusion, the present study suggests that deterioration of glucose homeostasis in women is associated with a greater impairment in myocardial glucose metabolism as compared with men. These findings may help to clarify the pathophysiological mechanisms by which hyperglycemia exceeding the diagnostic threshold of T2DM almost completely abolish the female protection from CVD.

## Data Availability

The datasets used and analysed during the current study are available from the corresponding author on reasonable request.

## Abbreviations

CVD: Cardiovascular disease
IDF: International Diabetes Federation
T2DM: Type 2 diabetes mellitus
PET: Positron emission tomography
MRGlu: Myocardial glucose metabolic rate
^18^F-FDG: ^18^F-Fluorodeoxyglucose
BMI: Body mass index
BP: Blood pressure
OGTT: Oral glucose tolerance test
FPG: Fasting plasma glucose
ADA: American Diabetes Association
NGT: Normal glucose tolerance
M_FFM_: Insulin-stimulated glucose disposal corrected for fat-free mass
FFM: Fat-free mass
PCARD: Tool specific for cardiac images analysis
VOI: Volume of interest
